# Distribution of ADAT-Dependent Codons in the Human Transcriptome

**DOI:** 10.3390/ijms160817303

**Published:** 2015-07-29

**Authors:** Àlbert Rafels-Ybern, Camille Stephan-Otto Attolini, Lluís Ribas de Pouplana

**Affiliations:** 1 Institute for Research in Biomedicine (IRB), Parc Cientific de Barcelona, C/Baldiri Reixac 10, 08028 Bacelona, Spain; E-Mails: albert.rafels@irbbarcelona.org (A.R.-Y.); camille.stephan@irbbarcelona.org (C.S.-O.A.); 2 Catalan Institution for Research and Advanced Studies (ICREA), Passeig Lluís Companys 23, 08010 Barcelona, Spain

**Keywords:** tRNA modification enzymes, ADAT2-ADAT3, codon degeneracy, tRNA gene copy number

## Abstract

Nucleotide modifications in the anticodons of transfer RNAs (tRNA) play a central role in translation efficiency, fidelity, and regulation of translation, but, for most of these modifications, the details of their function remain unknown. The heterodimeric adenosine deaminases acting on tRNAs (ADAT2-ADAT3, or ADAT) are enzymes present in eukaryotes that convert adenine (A) to inosine (I) in the first anticodon base (position 34) by hydrolytic deamination. To explore the influence of ADAT activity on mammalian translation, we have characterized the human transcriptome and proteome in terms of frequency and distribution of ADAT-related codons. Eight different tRNAs can be modified by ADAT and, once modified, these tRNAs will recognize NNC, NNU and NNA codons, but not NNG codons. We find that transcripts coding for proteins highly enriched in these eight amino acids (*ADAT-aa*) are specifically enriched in NNC, NNU and NNA codons. We also show that the proteins most enriched in *ADAT-aa* are composed preferentially of threonine, alanine, proline, and serine (*TAPS*). We propose that the enrichment in ADAT-codons in these proteins is due to the similarities in the codons that correspond to *TAPS*.

## 1. Introduction

The genetic code is degenerate, as the number of amino acids coded is smaller than the number of possible codons, and multiple codons can code for the same amino acid. Typically, the multiple codons that can correspond to a single amino acid are not equally abundant in the genome. This codon bias is a signature of genomes, and can vary widely from species to species.

In addition, the number of tRNAs with different anticodons is always smaller that the number of codons used in any species, because a single tRNA anticodon may pair with more than one codon. This is due to the fact that a higher pairing permissiveness exists between the third position of the mRNA codons and the first position of the tRNA anticodon. This is still known as wobble or degenerate pairing [1], although recent crystallographic data has shown that the ribosome enforces a Watson–Crick geometry even at the third position of the codon-anticodon interaction [2].

**Figure 1:**
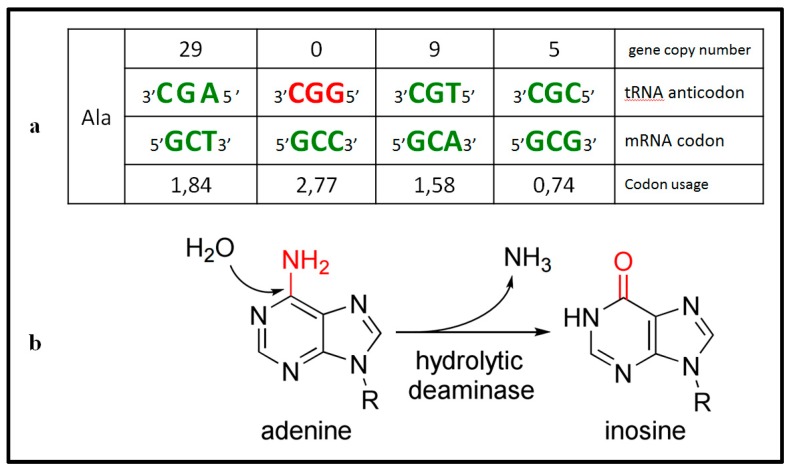
(**a**) Codon–anticodon relation for ADAT-related alanine amino acid. tRNA copy number and codon usage is shown for each pair, note that $tRNA_{GGC}^{Ala}$ do not exist in human (red nucleotides); (**b**) Hydrolytic deamination: Adenine is converted into an inosine throughout a hydrolytic deamination reaction.

We have recently shown that codon bias and tRNA gene copy number in eukaryotes were influenced by the emergence of heterodimeric adenosine deaminases acting on tRNAs (ADAT), which deaminate A34 to I34 in those tRNAs with ANN anticodons that decode threonine, alanine, proline, and serine (*TAPS*), and leucine, isoleucine, valine, and arginine (*LIVR*) [3] (Figure 1b). Here we will refer to these amino acids as *ADAT-aa*.

ADAT most likely evolved from the homodimeric bacterial adenosine deaminase TadA, which acts solely on $tRNA_{ACG}^{Arg}$ [4,5]. The emergence of ADAT was instrumental in the enrichment of genes coding for *TAPS* and *LIVR* tRNAs with ANN anticodons in eukaryotes. With the exception of $tRNA_{ACG}^{Arg}$, these tRNAs are virtually absent in bacteria and archaea [6]. The activity of ADAT effectively modifies the pool of tRNAs available for each codon, and aligns the correlation between codon usage and tRNA gene copy number in eukaryotes [3].

Inosine 34-modified tRNAs can “wobble” pair with A, C or U, and this solves the apparent riddle offered by the abundance of C-ended codons coding for *TAPS* and *LIVR* and the complete absence of the corresponding tRNAs with GNN anticodons in eukaryotes (Figure 1a) [7]. ADAT was first shown to be an essential enzyme in yeast, and has been later characterized in *Trypanosoma*, and *Arabidopsis* [5,8,9,10]. We have recently shown that, in *Homo sapiens*, the modification by ADAT of the eight cytoplasmic tRNAs, $tRNA_{IGC}^{Ala}$, $tRNA_{IGG}^{Pro}$, $tRNA_{IGU}^{Thr}$, $tRNA_{IAC}^{Val}$, $tRNA_{IGA}^{Ser}$, $tRNA_{ICG}^{Arg}$, $tRNA_{IAG}^{Leu}$ and $tRNA_{IAU}^{Ile}$, takes place predominantly in the nucleus, during the maturation process of these molecules [11].

Generally speaking, the study of the influence of anticodon modifications on the translation of specific codons has recently led to the realization that tRNA populations can act as a new layer of gene translation regulation through the modulation of their anticodon modification status, or through changes in the expression levels of different tRNA genes [12,13,14,15,16,17]. In the case of ADAT, and despite its importance in the evolution of eukaryotic genomes, little is known about its potential role in translation regulation [12]. Here we present the first analysis of the distribution of ADAT-related codons in the human transcriptome, and computationally characterize the proteins most rich in *ADAT-aa*.

To try to identify those polypeptides whose translation is more likely to be influenced by ADAT activity, we have first classified the human proteome according to the abundance of *ADAT-aa* in each protein. We have used two different methods to determine the distribution of *TAPS*-, and *LIVR*-coding triplets in the human genome: a *half-gene* analysis, and a *running-window* approach. Those transcripts with a significantly increased proportion of these triplets have been analyzed for their composition in ADAT-preferred codons, to test if these are enriched with respect to G-ended codons in these proteins.

We show that, in general, ADAT codons (those that can be recognized by tRNAs modified by ADAT) are generally preferred to G-ended codons (not recognizable by ADAT) in the human genome. Moreover, this preference increases in proteins enriched in *ADAT-aa*. Interestingly, although we included both *TAPS* and *LIVR* in the search for proteins highly enriched in ADAT-related codons, we find that the most biased human protein sequences in this regard are only enriched in *TAPS*.

Coherently, in these sequences only the triplets for *TAPS* are enriched for ADAT-dependent codons, indicating that the activity of ADAT may be important for the translation of gene regions coding for long stretches of *ADAT-aa*. We argue that this enrichment may be explained by the fact that codons for *TAPS* occupy a close position in the genetic code, where all of them share the same second base.

More importantly, our results hint at the possibility that the emergence of ADAT allowed eukaryotic cells to produce highly repetitive protein sequences that bacterial or archaeal ribosomes may be unable to translate due to the absence of I34-containing tRNAs in these organisms.

## 2. Materials and Methods

### 2.1. Definitions

*ADAT-aa* are defined as those amino acids that are charged to tRNAs that can be modified by ADAT (Thr, Ala, Pro, Thr, Ser (*TAPS*), and Leu, Ile, Val, and Arg (*LIVR*)). *C* is defined as the set of all the 64 codons. *A* is defined as the total set of codons that code for any of the *ADAT-aa*, and corresponds to the 37 codons present in Figure S6. *D* is defined as the subset of 24 codons of *A* that are recognized by tRNAs modified by *ADAT* at position 34 (Figure S6 codons that can “wobble” pair with I34 anticodons). For a given region *t* of a coding sequence, the amount of codons in *t* that belongs to *C*, *A* or *D* is defined as *c*(*t*), *a*(*t*) or *d*(*t*), respectively. *ADAT stretch* are those regions that have a high value for *a*(*t*)/*c*(*t*) compared with the rest of the transcriptome. We will define the stretch in more detail in the next section.

### 2.2. Human Transcriptome Retrieval

We have analyzed 28,870 human Coding Sequences (CDSs) that conform the human transcriptome. All these sequences have been downloaded from the Consensus CDSs (CCDS) project [18]. Only CDSs with start codon, stop codon and a number of nucleotides multiple of 3 were used for our analysis. Only 48 CCDSs were eliminated.

### 2.3. Identification of Stretches by the Halves-Gene Method

To carry on this analysis we developed the *Halves-gene method*. Each CDSs of the human transcriptome is recursively divided into halves and for each region *a*(*t*) is calculated (Figure S1). We divided each CDS until sections of ~15 codons were reached. When the region to be divided had an odd number of codons, the first half was assigned one codon more that the second (Figure S1). Note that this method has the disadvantage that each CDS is represented several times but with different lengths. However, as all the CDSs are equally treated, there is no bias in the final data.

The variability in Figure S7 was measured by Interquartile Range (IQR). IQR is equal to the difference between the 1st and the 3rd quartiles (*IQR* = *Q*_3_ − *Q*_1_). The density plot in Figure S5 was computed using the *smoothScatter* function in *graphics* package for R. Multiple linear regression in Figure S5 was computed using the *segmented* package for R with seeds 0.3 and 0.7 [19] and the slopes were obtained with the function *slope*.

### 2.4. Identification of Stretches by the Running Windows Method

**Figure 2:**
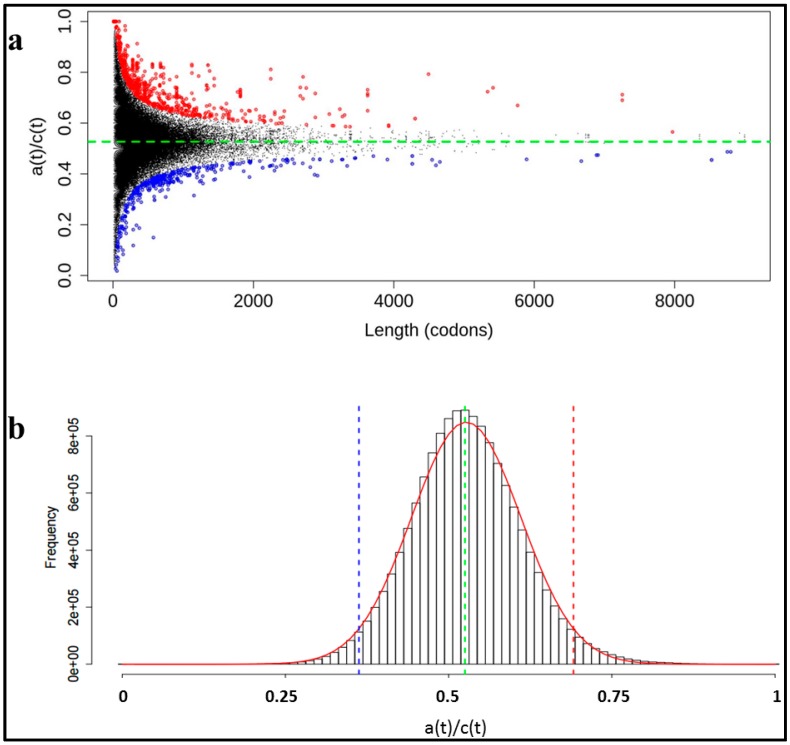
(**a**) Distribution of the human transcriptome using the *Halves method* to study the enrichment in *A codons*. The dashed green line shows the mean of *A codons* in the human transcriptome (0.527). Black dots correspond to all the regions obtained with this method (see Section 2.3). Red circles correspond to the enriched regions (2666). Blue circles correspond to the unenriched regions (412). Both regions are calculated supposing a binomial distribution with *p*-value <10^−11^; (**b**) Running Windows Method distribution. *a*(*t*) enrichment for all the windows. Red line is the normal distribution following this histogram. Blue and red dashed lines show where the tails of the distribution represents a 5%. Green dashed line shows the mean.

**Figure 3:**
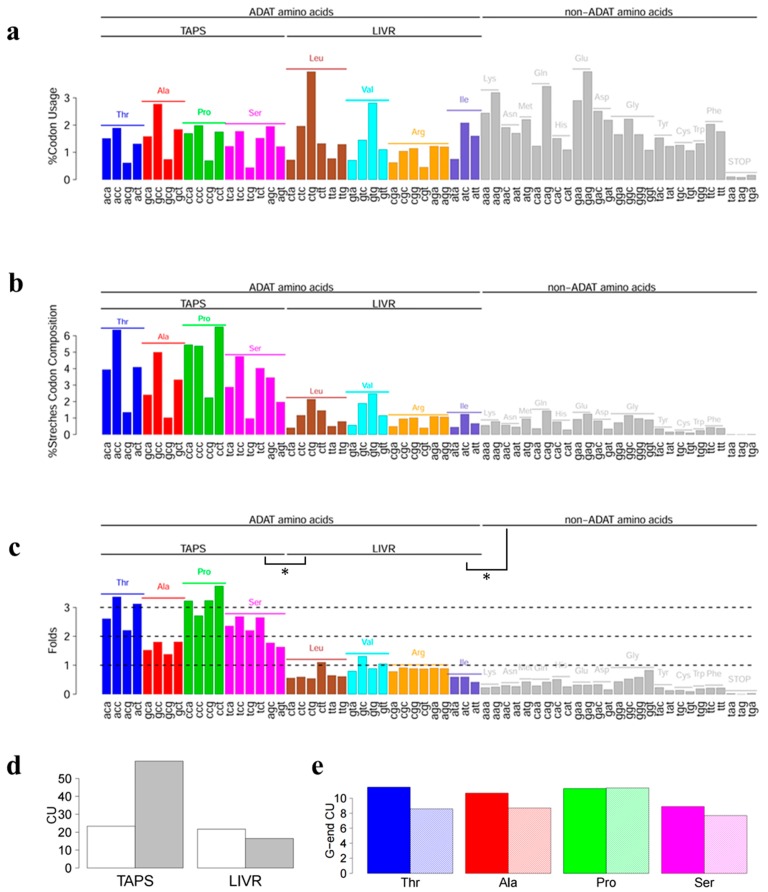
(**a**) Codon usage of the human transcriptome [24]; (**b**) Codon usage for *ADAT stretches*. *A codons* are colored with a different color for each ADAT amino acid. (**a**,**b**) Each percentage is measured with respect to the total of codons, thus all the bars sums 100%; (**c**) Fold increase of *ADAT stretches* (**b**) normalized by human codon usage (**a**). There are significant differences (see asterisks *) between sets of *TAPS* and *LIVR* (*p*-value = 10^−7^), and sets of *LIVR* and the *non-ADAT amino acids* (*p*-value = 5 × 10^−8^). One-tail Wilcoxon test was applied in both cases with confidence level 0.95; (**d**) Codon usage for *TAPS* or *LIVR* codons in the human transcriptome (white) or in the *ADAT stretch* regions (grey); (**e**) Codon usage for G-ended codons for *TAPS* in the human transcriptome (colored) or in the *ADAT stretches* (dashed colored).

The False Discovery Rate (*FDR*) is the ratio between the expected values and the obtained values (red line, and histogram bars respectively in Figure 2b). Wilcoxon test (Figure 3) was computed using *stats* package for R.

To study in more detail the presence of *stretches* of *A codons* in the human transcriptome, we applied the *Running Windows method* (Figure S4) based on software that applies similar methodologies [20,21,22]. For each CDS of the human transcriptome a window (fragment of the sequence with a fixed size) slides codon by codon from the beginning to the end of the sequence (Figure S4a). For each window, *a*(*t*) is calculated and represented with respect to its location (Figure S4b). To fix the window size we took advantage of the previous method (*Halves-gene method*) to find a region length as small as possible but with a low variability (Figure S7). We fixed a window length of 80 codons because it has a low variability (IQR ~40%) and because this length approximates the average size of single protein domains [23]. We arbitrarily limited future analyses to windows enriched in *A codons* with FDR <0.2, *i.e.*, those windows with *a*(*t*) comprised in the interval 67–80 (Figure S3). We define an *ADAT stretch* as those regions corresponding to a window, or a set of consecutive windows with this enrichment (Figure S4). Two (or more) windows are considered consecutive if the intersection between them in the cognate CDS is not void. This corresponds to 560 sequences containing stretches (≥80 codons) of *A codons* in 242 different human genes.

## 3. Results and Discussion

### 3.1. Identification of Human Proteins Highly Enriched in ADAT-aa

Using the two strategies described in Section 2, we identify those transcript sequences more enriched in *A codons*, which correspond to protein regions that are highly enriched in *ADAT-aa* compared to the rest of the proteome. Figure 2a shows the distribution of *a*(*t*)/*c*(*t*) of all the fragments analyzed, as a function of their sequence length using the *Halves-gene method* (Figure S1). The dist ribution is centered on the mean of *A codons* for the human transcriptome (0.527, dashed green line). The variability in the *y*-axis decreases as the length of the fragments increases. We detect a number of sequences (red and blue points) that significantly deviate from an expected random distribution. This behavior is expected because of the non-random nature of the genome.

Supposing that all the samples follow a binomial distribution, we identified outliers with a *p*-value <10^−11^. There are 2666 samples enriched in *A codons*, and 412 depleted of *A codons*. Therefore the distribution of the outliers is not well-balanced (Figure S2), with a tendency of the CDSs to create regions highly enriched in *A codons*.

### 3.2. Stretches of A codons Are Composed Preferentially by Triplets Coding for TAPS

The *Running Windows method* gives a different distribution compared to the *Halves-gene method* due to the different nature of the data (Figure S4, Section 2). Figure 2b shows the distribution of all the windows for the whole transcriptome, based in the abundance of *A codons.* The most frequent value is at *a*(*t*)/*c*(*t*) = 0.525 (green line). Note that in this method *c*(*t*) have a constant value of 80 codons. The mean for all the windows is at *a*(*t*)/*c*(*t*) = 0.526 and the codon usage for *A codons* is 0.527. If normality of the data is assumed (red line), 95% of windows are [24] comprised between the region [0.362, 0.7] (region between blue and red lines). The windows outside of this central region are either depleted or enriched in *A codons*. The number of enriched windows is 5.2 × 10^5^ while the number of unenriched windows is 2.7 × 10^5^. Note that, when comparing the two tails, *FDR* for the enriched tail is always lower than the *FDR* for the unenriched tail (Figure S3), thus the distribution is not symmetric and indicates again a preference in the human transcriptome for sequences enriched in *A codons*.

Figure 3b shows the composition of individual codons for all the *ADAT stretches*, and Figure 3a shows the codon usage for the whole human transcriptome. Surprisingly, not all the *A codons* are equally enriched in *ADAT stretches*. Codons for *TAPS* strongly predominate over codons for *LIVR* (*p*-value = 10^−7^) (Figure 3d; Table S1), indicating that the enrichment of ADAT-dependent codons is not uniform, and that the concentration of *TAPS* can reach much higher values in proteins than the concentration of *LIVR*.

Figure 3c shows the fold change for codon composition comparing the stretches with the rest of the genome. Each residue in the *TAPS* group reaches enrichments of more than two-fold with respect to the mean, with threonine and proline reaching almost three-fold increases. Strikingly the concentration of *LIVR* codons decreases in the *ADAT stretches* (Figure 3d; Table S2). Finally, G-ended codons for *TAPS* are significantly decreased in the *ADAT stretches*, with the sole exception of Pro, which remains stable (Figure 3e; Table S3).

### 3.3. ADAT Stretches Are Composed Preferentially of D codons

**Figure 4:**
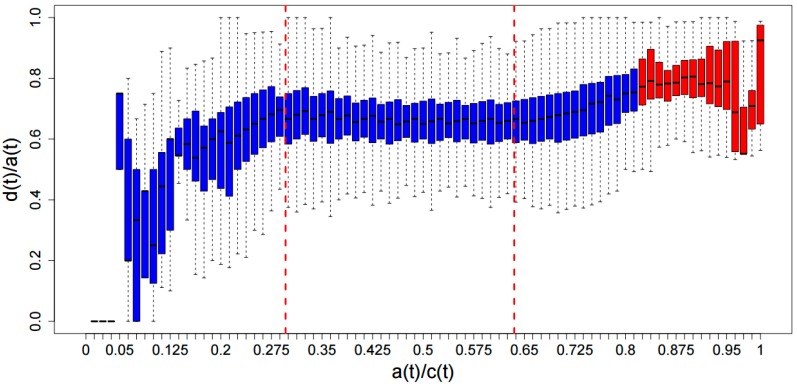
Graph of boxplots for concentration of *A codons*, *a*(*t*)/*c*(*t*), *versus* the relative concentration of *D codons*, *d*(*t*)/*a*(*t*). Blue boxes correspond to current windows, whereas the red ones correspond to the *ADAT stretches*. Multiple linear regression based on the mean values found two breakpoints at *a*(*t*)/*c*(*t*) 0.296 and 0.635 (dashed red lines) (See Figure S5 for more details).

We asked whether *ADAT stretches* would be significantly enriched in *D codons*, and depleted of G-ended codons. To carry out this analysis we calculated the relative concentration of *D codons*, that is, *d*(*t*)/*a*(*t*) for all the samples. Figure 4 shows a boxplot graph of *d*(*t*)/*a*(*t*) for all the windows (blue bars) and all the stretches (red bars) belonging to the respective interval in *a*(*t*)/*c*(*t*). Those boxplots that correspond to an stretch are plotted in red located at the region *a*(*t*) > 67. Figure S5 shows a density plot for all the samples where the points correspond to the means in the boxplot intervals graph, and the black lines correspond to a multiple linear regression based on the mean values. Two breakpoints can be seen when *a*(*t*)/*c*(*t*) is 0.296 and 0.635 (Figure 4, dashed red lines). The behavior of the data is well differentiated and can be divided into three regions. The region *a*(*t*)/*c*(*t*) in [0, 0.296] comprises only a 0.27% of the of windows and therefore the linear regression is not considered. The region *a*(*t*)/*c*(*t*) in [0.296, 0.635] comprises 89.48% of windows and the linear regression is essentially flat (slope −0.07 ± 0.03), indicating a non-dependence between *a*(*t*)/*c*(*t*) and *d*(*t*)/*a*(*t*)*.* Finally, *a*(*t*)/*c*(*t*) in [0.635, 1] contains 10.25% of windows with a slope of 0.52 ± 0.03, showing that there is a clear dependence between *a*(*t*)/*c*(*t*) and *d*(*t*)/*a*(*t*) in this region that contains both the *ADAT stretches* (red boxes) for the highest values and non-stretched regions (blue boxes).

## 4. Conclusions

**Figure 5:**
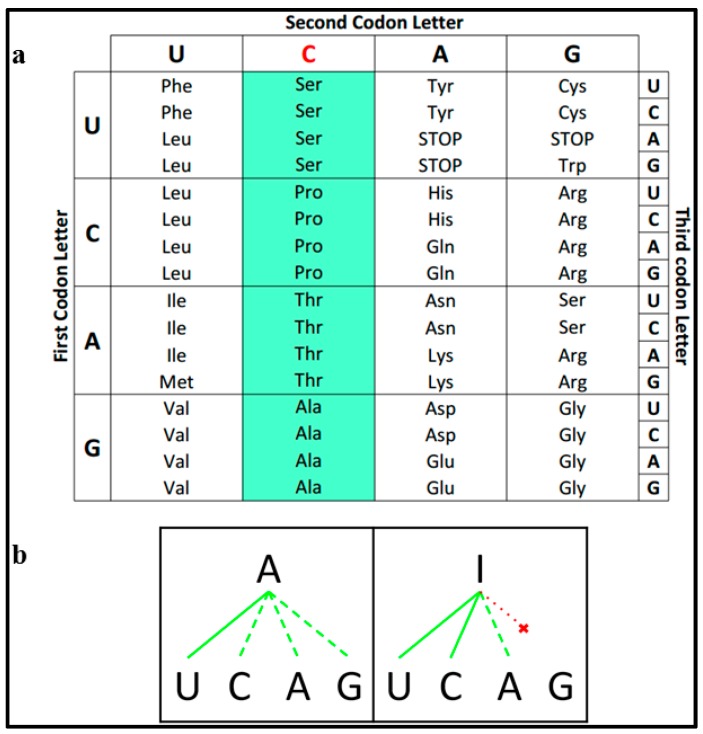
(**a**) Codon usage table. Highlighted in green are all the codons that translate for *TAPS* that share a common C in their second position (red); (**b**) Schematic representation of base pairing between Adenine (or Inosine) and the rest of the classical bases. Continuous green lines indicate preferred pairings and dashed green lines indicate poor pairings. Dashed red line indicates no pairing.

The biological significance of inosine at position 34 of anticodons is generally linked to the pairing ability of this nucleotide, which allows harboring anticodons to recognize codons with C, U and A (Figure 5b) [4]. This is in contrast to adenosine, which is supposed to favor a Watson–Crick interaction with uridine, although several reports demonstrate that adenosine is capable of pairing with any base in the third position of the codon (Figure 5b) [4,25]. Thus, it remains unclear whether inosine is widely used in eukaryotes to restrict or expand the pairing capacity of A34 containing codons [4,26,27,28].

It is clear that inosine is important to balance codon usage and tRNA gene copy number in eukaryotes, and that highly translated genes in these species tend to be enriched in ADAT-dependent codons [3]. In the light of the growing realization of the regulatory role that modification enzymes, and fluctuations in tRNA populations, play in the regulation of specific gene programs, it is important to determine if inosine is also involved in the regulation of gene expression levels.

To start addressing this question we have begun to characterize how ADAT-dependent codons are distributed in eukaryotic proteomes. This initial analysis is required to try to identify sections of the transcriptome potentially more dependent on the ADAT activity levels. Our approach has been, first, to screen the complete human transcriptome and classify its genes on the basis of the abundance of *ADAT-aa*. Using this initial curation, we have identified those transcripts whose proportion of codons for such *ADAT-aa* is significantly enriched, and we have used this subset of sequences to determine the relative enrichment of each *ADAT-aa*, and the variation in *D* and G-ended codons in this group of sequences with respect to the whole transcriptome.

Our results show, first, that the human transcriptome is biased towards proteins enriched in *ADAT-aa*. Interestingly, the majority of these proteins are specifically enriched in *TAPS*, but not in *LIVR*. Physicochemical parameters specific to these residues may explain why *TAPS* can reach higher relative frequencies than *LIVR* in human proteins. At the same time, functional features of proteins rich in *TAPS* must have driven the selection of these extremely biased protein sequences.

Our data also shows that the more enriched in *ADAT-aa* a region is, the higher its tendency to use *D codons* instead of G-ended codons. Therefore, transcripts coding for stretches of *ADAT-aa* are composed preferentially by *D codons*, and this composition increases with the length and quality of the stretch. This observation confirms the notion that ADAT-modified tRNAs are preferred in eukaryotic translation, and suggests that the selective force behind this selection is increased when the frequency of *ADAT-aa* rises.

The reason why stretches of TAPS amino acids are enriched in ADAT-dependent codons remains to be determined. In this regards, it is interesting to notice that the four amino acids that are enriched in the stretches (threonine, alanine, proline, and serine) are all coded by four-box codon sets that share the same second base (C) (serine is also coded by two additional codons) (Figure 5a). Thus, the selectivity between these four codon sets depends only upon recognition of the first codon base. Under these circumstances, it is possible that the proposed higher selectivity of I over A may be preferred to minimize the possibility of decoding errors, particularly in highly repetitive transcript regions such as the stretches identified in our analysis.

An important corollary of our analysis is the potential role of anticodon modifications in allowing ribosomal protein synthesis machineries to access new protein sequence spaces. Several examples of codon and amino acid compositions are known that impair ribosomal functional and, in some cases, require additional cofactors to allow the ribosome to progress through these regions [29,30,31,32]. It is similarly conceivable that certain highly repetitive transcript sequences may be inaccessible to ribosome processing unless new functional improvements that increase efficiency or selectivity can be found. The selection of modified bases, such as inosine, that possibly allow species to synthesize proteins previously unavailable may be a major driving force in speciation. A detailed evolutionary analysis of ADAT function in the eukaryotic lineage will contribute to test this hypothesis.

## Supplementary Materials

Supplementary materials can be found at http://www.mdpi.com/1422-0067/16/08/17303/s1.
