# Supplementary material for: Distribution of ADAT-Dependent Codons in the Human Transcriptome
Source: Int J Mol Sci. 2015 Jul 29;16(8):17303–14. doi: 10.3390/ijms160817303 (PMC4581194; doi:10.3390/ijms160817303)
Supplement: Supplementary file 1 [file ijms-16-17303-s001.pdf]

Supplementary Information

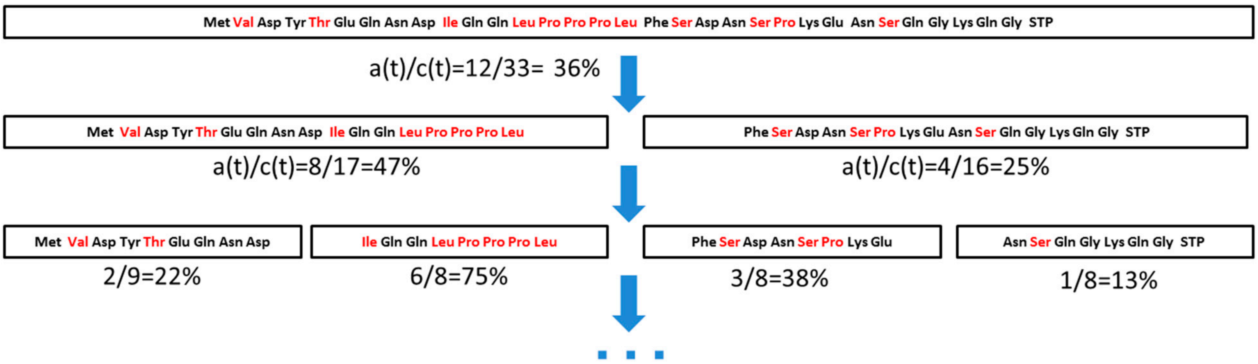

Figure S1. *Halves-gene* method layout. Each sequence is divided into halves recursively (blue arrows) until the regions are small enough. For each region $a(t)/c(t)$ is calculated. Red amino acids correspond to *A codons*.

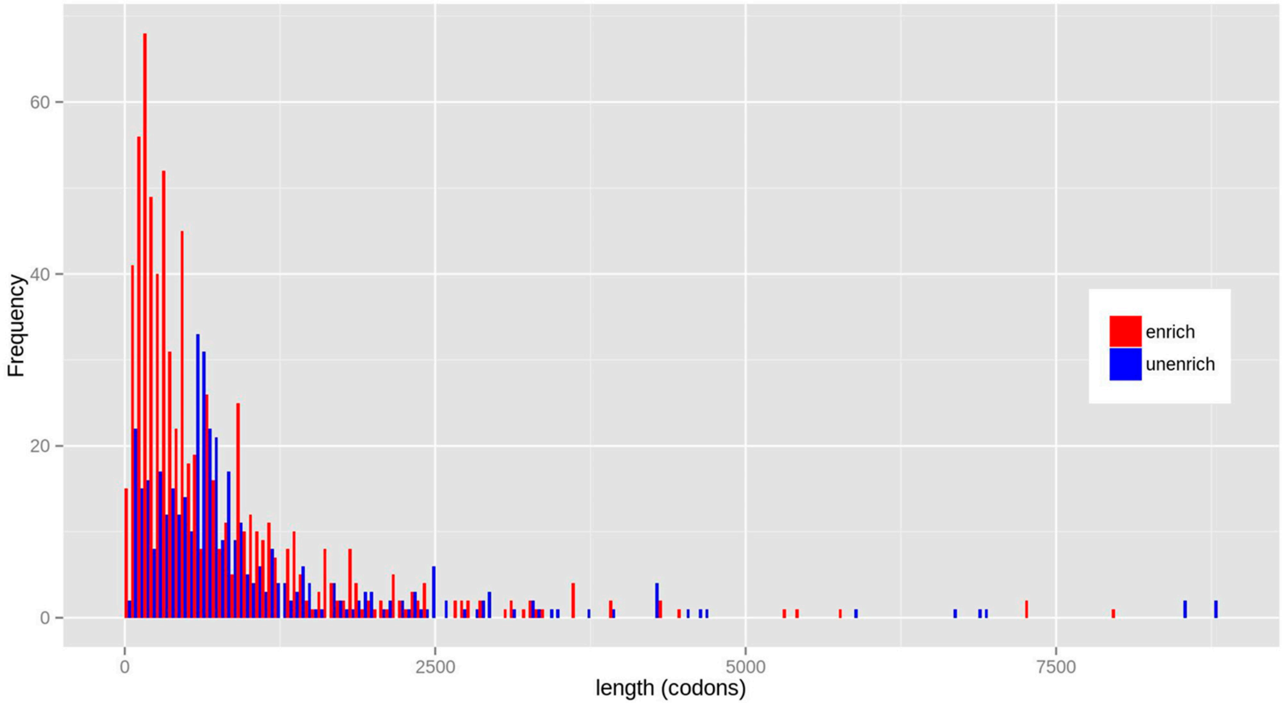

Figure S2. Histogram comparing the enriched (red) and the unenriched (blue) outliers from Figure 2a.

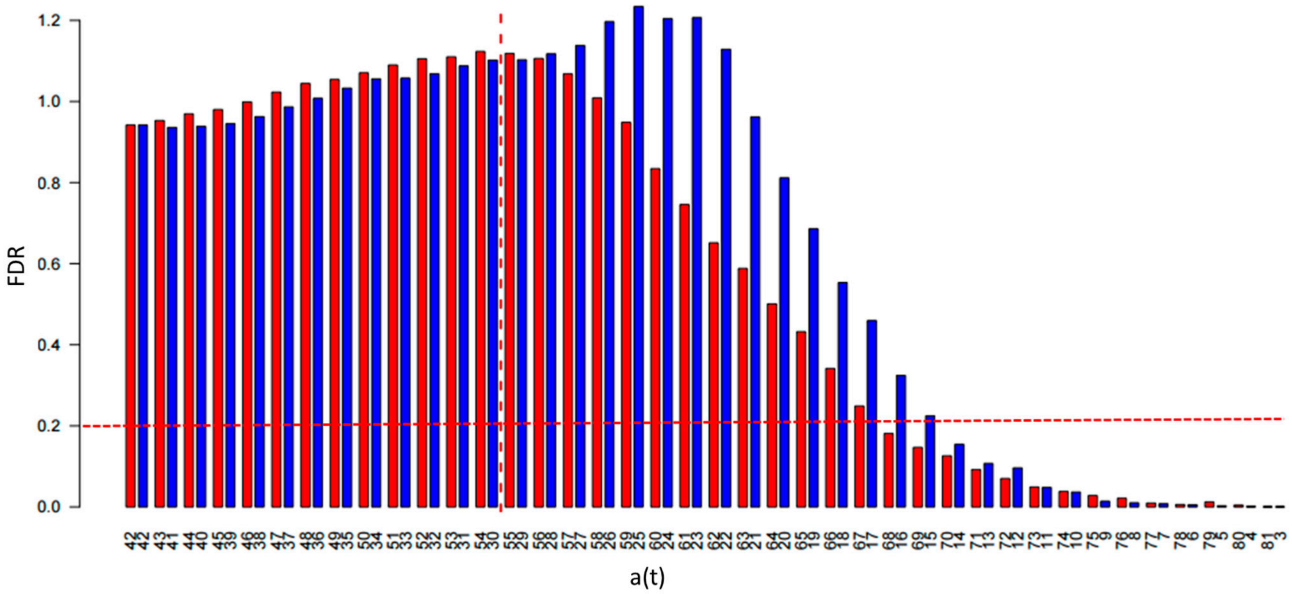

Figure S3. Histogram comparing the False Discovery Rate (FDR) symmetry of Figure 2b. Tails of 5% (from the horizontal dashed red line to the right) for enriched windows (red bars) and unenriched windows (blue bars). *ADAT stretches* are considered for those windows with *FDR* < 0.2 (vertical dashed red line), or equivalently $a(t)$ > 67.

a

b

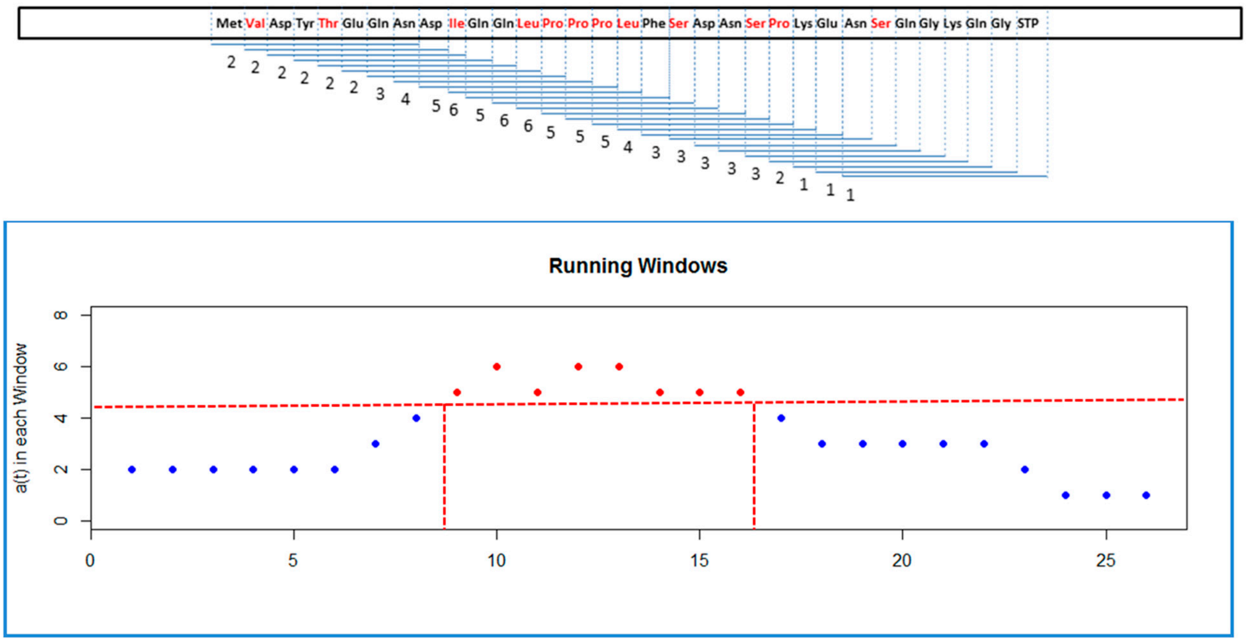

Figure S4. *Running-windows* layout (**a**) Scheme of action for this method. For each sequence the window slides codon by codon from the beginning to the end (dashed blue lines) and $a(t)$ is calculated; (**b**) Distribution of $a(t)$ for each window, the enriched windows correspond to the highest $a(t)$ values. In this example, there is one single stretch (between horizontal dashed red lines) that consists of eight consecutive enriched windows (red points). Red amino acids correspond to *A codons*.

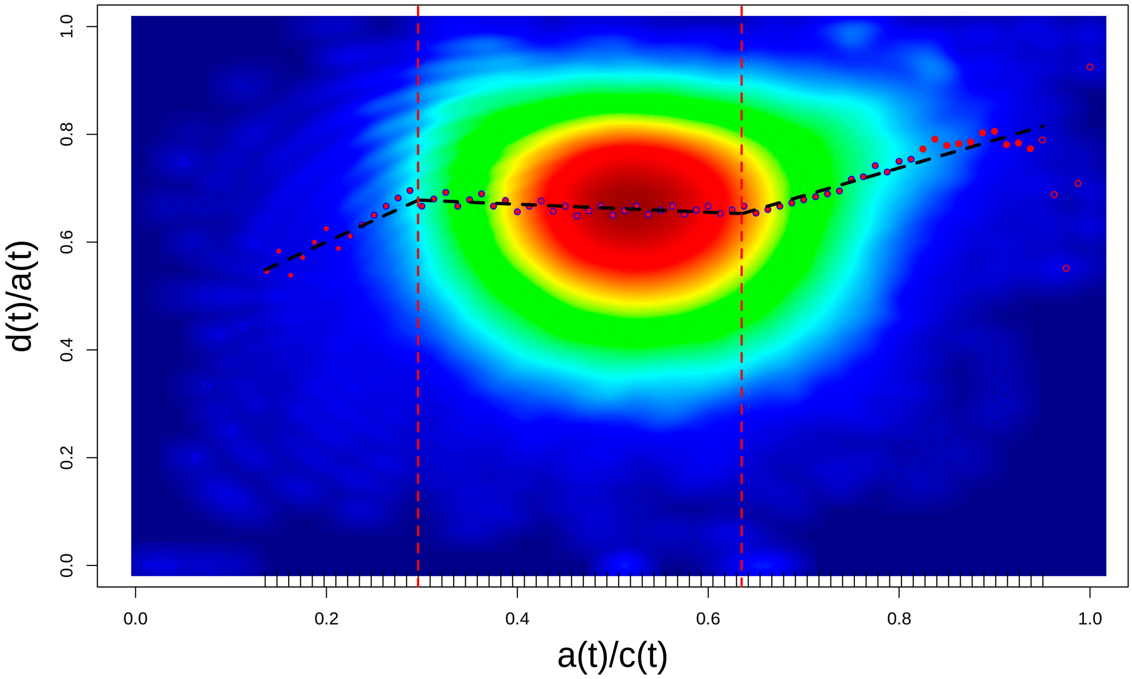

Figure S5. Density plot between $a(t)/c(t)$ and $d(t)/a(t)$. The dots correspond to the mean values in Figure 4 and the dashed black lines correspond to a multiple linear regression based on these values. There are two breakpoints at $a(t)/c(t)$ equal to 0.296 and 0.635 (dashed red lines).

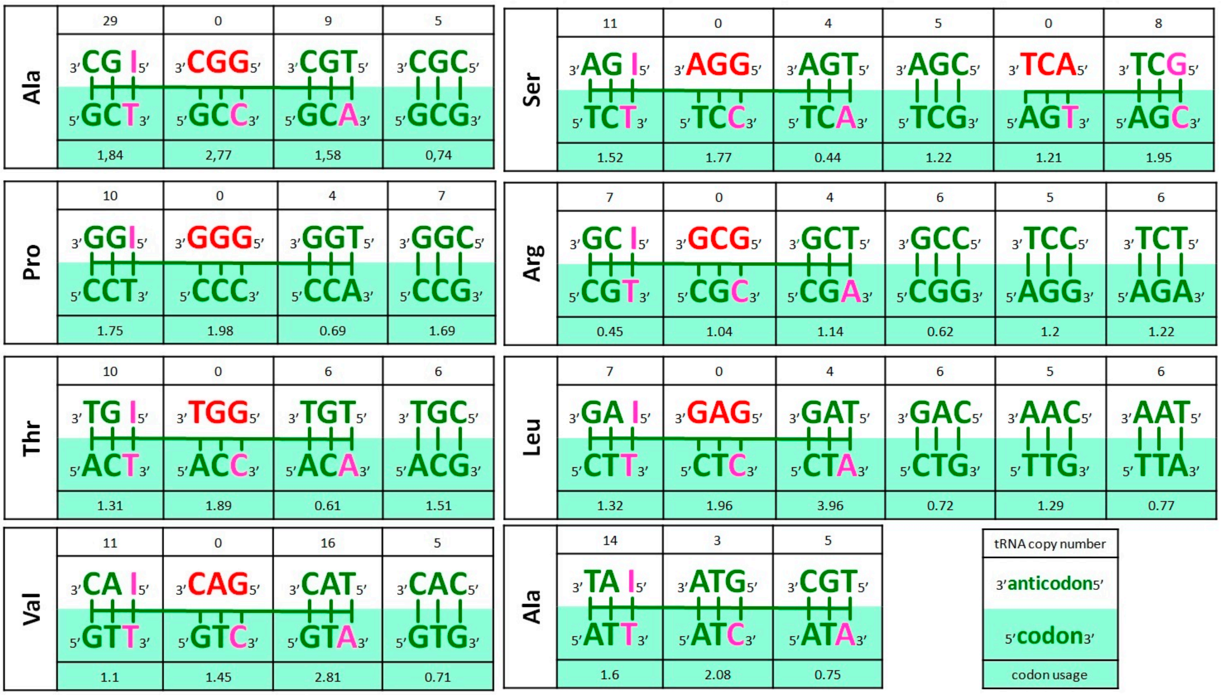

Figure S6. Codon-anticodon relationships for *ADAT-aa*. tRNA copy number and codon usage is shown for each pair, as indicated in bottom-right legend. Inosine 34 and those nucleotides recognized throughout "wobble" pairing are depicted in pink. Green lines shows which codon-anticodon pairings takes place. Anticodons depicted in red do not exist in human genome.

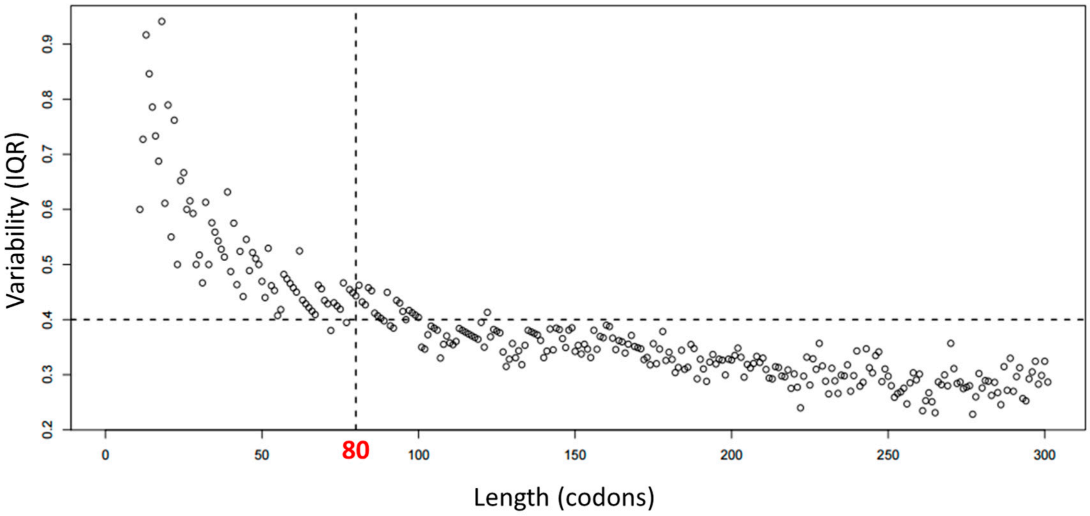

Figure S7. Variability corresponding to Figure 2a calculated by Interquartile Range (IQR).

Table S1. Data corresponding to Figure 3a–c. *aa*: amino acids, *cod*: codons, *CU*: codon usage, *Str*: *ADAT stretches* dataset, *All*: Human transcriptome dataset, *fold*: rate Str/All. Each percentage is measured with respect to the total of codons, thus Str and All columns add up to 100%.

aa	cod	CU		fold
		Str	All	
Thr	aca	3.94	1.51	2.61
	acc	6.36	1.89	3.37
	acg	1.35	0.61	2.21
	act	4.09	1.31	3.12
Ala	gca	2.4	1.58	1.52
	gcc	4.99	2.77	1.80
	gcg	1.02	0.74	1.38
	gct	3.32	1.84	1.80
Pro	cca	5.45	1.69	3.22
	ccc	5.37	1.98	2.71
	ccg	2.23	0.69	3.23
	cct	6.54	1.75	3.74
Ser	tca	2.88	1.22	2.36
	tcc	4.75	1.77	2.68
	tcg	0.97	0.44	2.20
	tct	4.03	1.52	2.65
	agc	3.46	1.95	1.77
	agt	1.97	1.21	1.63

aa	cod	CU		fold
		Str	All	
Leu	cta	0.4	0.72	0.56
	ctc	1.17	1.96	0.60
	ctg	2.14	3.96	0.54
	ctt	1.45	1.32	1.10
	tta	0.5	0.77	0.65
	ttg	0.79	1.29	0.61
Val	gta	0.57	0.71	0.80
	gtc	1.89	1.45	1.30
	gtg	2.48	2.81	0.88
	gtt	1.15	1.1	1.05
Arg	cga	0.48	0.62	0.77
	cgc	0.95	1.04	0.91
	cgg	1.01	1.14	0.89
	cgt	0.39	0.45	0.87
	aga	1.1	1.22	0.90
	agg	1.06	1.2	0.88
Ile	ata	0.44	0.75	0.59
	atc	1.24	2.08	0.60
	att	0.67	1.6	0.42

aa	cod	CU		fold
		Str	All	
Lys	aaa	0.55	2.44	0.23
	aag	0.79	3.19	0.25
Asn	aac	0.57	1.91	0.30
	aat	0.45	1.7	0.26
Met	atg	0.94	2.2	0.43
Gln	caa	0.35	1.23	0.28
	cag	1.43	3.42	0.42
His	cac	0.77	1.51	0.51
	cat	0.28	1.09	0.26
Glu	gaa	0.92	2.9	0.32
	gag	1.25	3.96	0.32
Asp	gac	0.83	2.51	0.33
	gat	0.33	2.18	0.15
Gly	gga	0.71	1.65	0.43
	ggc	1.16	2.22	0.52
	ggg	0.96	1.65	0.58
	ggt	0.89	1.08	0.82
Tyr	tac	0.35	1.53	0.23
	tat	0.15	1.22	0.12
Cys	tgc	0.17	1.26	0.13
	tgt	0.1	1.06	0.09
Trp	tgg	0.24	1.32	0.18
Phe	ttc	0.43	2.03	0.21
	ttt	0.38	1.76	0.22
STOP	taa	0	0.1	0.00
	tag	0	0.08	0.00
	tga	0	0.16	0.00

Table S2. Data corresponding to Figure 3d. Same notation as in Table S1. *4box* means that for the amino acids Ser, Leu and Arg, only the 4box XXN codons were taken into account.

	CU	
	Str	All
**TAPS (4box)**	59.69	23.31
**LIVR (4box)**	16.43	21.71

Table S3. Data corresponding to Figure 3e. Same notation as in Table S2. *G*: G-ended codon for the corresponding amino acid. %*G*: ratio *G*/*4box*.

aa	cod	CU		aa	cod	CU	
		Str	All			Str	All
**Thr**	**4box**	15.74	5.32	**Leu**	**4box**	5.16	7.96
	**G**	1.35	0.61		**G**	2.14	3.96
	**%G**	8.58	11.47		**%G**	41.47	49.75
**Ala**	**4box**	11.73	6.93	**Val**	**4box**	6.09	6.07
	**G**	1.02	0.74		**G**	2.48	2.81
	**%G**	8.70	10.68		**%G**	40.72	46.29
**Pro**	**4box**	19.59	6.11	**Arg**	**4box**	2.83	3.25
	**G**	2.23	0.69		**G**	1.01	1.14
	**%G**	11.38	11.29		**%G**	35.69	35.08
**Ser**	**4box**	12.63	4.95	**Ile**	**4box**	2.35	4.43
	**G**	0.97	0.44		**G**	-	-
	**%G**	7.68	8.89		**%G**	-	-
